# PCR-Stop analysis as a new tool for qPCR assay validation

**DOI:** 10.1038/s41598-018-26116-x

**Published:** 2018-05-29

**Authors:** Anna Kristina Witte, Patrick Mester, Susanne Fister, Beate Süß, Martin Wagner, Peter Rossmanith

**Affiliations:** 1 Christian Doppler Laboratory for Monitoring of Microbial Contaminants, Department of Veterinary Public Health and Food Science, University of Veterinary Medicine, Veterinaerplatz 1, 1210 Vienna, Austria; 2 Institute of Milk Hygiene, Milk Technology and Food Science, Department of Veterinary Public Health and Food Science, University of Veterinary Medicine, Veterinaerplatz 1, 1210 Vienna, Austria

## Abstract

Progressively more qPCR assays have been developed in recent years in numerous fields of application. These assays are routinely validated using calibration curves, but essential validation *per se* such as Poisson analysis is frequently neglected. However, validation is crucial for determination of resolution and quantitative and qualitative limits. The new test method PCR-Stop analysis presented in this work investigates assay performance during initial qPCR cycles. PCRs with one to five pre-runs are performed while the subsequent main qPCR runs reflect pre-run replication rates. Ideally, DNA doubles according to pre-runs, there is no variation between replicates and qPCR starts immediately at the first cycle with its average efficiency. This study shows two exemplary qPCR assays, both with suitable calibration curves and efficiencies. We demonstrated thereby the benefits of PCR-Stop analysis revealing quantitative and qualitative resolution of both assays, the limits of one of those assays and thus avoiding misinterpretations in qPCR analysis. Furthermore, data displayed that a well performing assay starts indeed with its average efficiency.

## Introduction

The *quantitative polymerase chain reaction* (qPCR) was first described in the 1990s^1,2^ and since then it has become a popular method in a wide field of applications. The method allows for quantification of DNA over a broad range using labelled probes or fluorescence dyes (summarized by Bonneta^3^). With each cycle, including steps for DNA denaturation, primer annealing and DNA extension (or a combined annealing-extension step), target DNA theoretically doubles, and consequently the fluorescence signal increases. The cycle that reaches the fluorescence threshold and is used for quantification, is customarily designated Ct (threshold cycle) or Cq (quantification cycle)^4^.

The past two decades have witnessed the development of numerous qPCR assays with differences in their performances. Advantages proposed include low running costs, short execution times and, with probe-based assays, inherent confirmation due to sequence specificity of the probe. However, although the method is conceptually simple, the execution must not be performed careless^5^. It is a highly sensitive method and thus, information of assay performance is extremely relevant. Moreover, qPCR represents an enzymatic assay and therefore it should be validated *per se*, according to the principles of organic chemistry^6,7^. Thus, inherent real performance parameters should be tested in each new qPCR to display the quality of the amplification reaction in a separate process. The main performance parameters are qualitative and quantitative limits and quantitative resolution.

Conventionally qPCR validation is performed by *Equivalence Partitioning Analysis* based on calibration curves using the Comparative Threshold Method (Ct-method^7^). Calibration curves are based on standard DNA samples starting from ten DNA molecules to >10^10^ copies^8^. PCR amplification efficiency is determined from these calibration curves and is subsequently used to determine the assay performance parameter. Briefly, when the PCR product doubles during each cycle the efficiency is theoretically 100% (or 1)^4^. The respective correlation coefficient Rsq consequently indicates the linearity of the PCR reaction^9^ and the precision of the pipetting process. Thereby, a value of 1.0 indicates perfectly linear amplification. This may result in seemingly satisfying efficiencies, but a perfectly linear amplification does not reflect automatically a good efficiency. Moreover, efficiency (and Rsq) reflects only a small statistical sample (the standard DNA samples). Consequently, this method provides limited main performance parameters information.

Sufficient information of main performance parameters can be obtained by testing the qPCR using Poisson analysis which reveals quantitative and qualitative resolution in the *Boundary Limit Area* (<10 ITMN (initial target molecule number))^6,7^. Validations based on Poisson distribution have steadily grown in popularity and applicability over recent years^10–12^. Nevertheless, it operates within the range 1 to <10 ITMN of the initial DNA template. Therefore, assays comprising a detection limit above ten ITMN cannot be validated for quantitative resolution or qualitative detection boundaries using this algorithm. Furthermore, it provides information concerning qualitative resolution, but it contributes no information about the actual efficiency of the amplification reaction. However, knowing whether efficiency is constant from beginning and in accordance with the average efficiency is crucial for analyzing qPCR performance.

We present here a new *Boundary Limit Analysis* assay validation tool, *PCR-Stop analysis*, which reflects assay performance during initial qPCR cycles. It operates in the range >10 ITMN, demonstrates two-fold resolution and consequently quantitative resolution and the quantitative limit of the assay. Moreover, the evaluation of efficiency is based on actual precision of the amplification reaction independent of statistical analysis of a calibration curve. Therefore, it provides sufficient information by means of main performance parameters, especially the quantitative resolution of the method in the range of >10 ITMN. Hence, the method should be performed in combination with Poisson analysis (<10 ITMN) for thorough assay validations. Improved knowledge of assay performance thus anticipates more consistent and reliable results. Validation with the PCR-Stop analysis should be especially interesting for relative gene expression analysis, especially when using the comparative Ct method (2^−ΔΔCt^) where the amplification efficiency is essential and which is widely used^13^.

In providing exemplary data with two assays, newly generated data can be compared, thereby facilitating qPCR assay validation. One of the assays is excellent in performance while the other showed equivalent calibration curves with similar efficiencies but revealed deficiencies in PCR-Stop analysis.

## Results

### Intention and execution of PCR-Stop analysis

**Figure 1:**
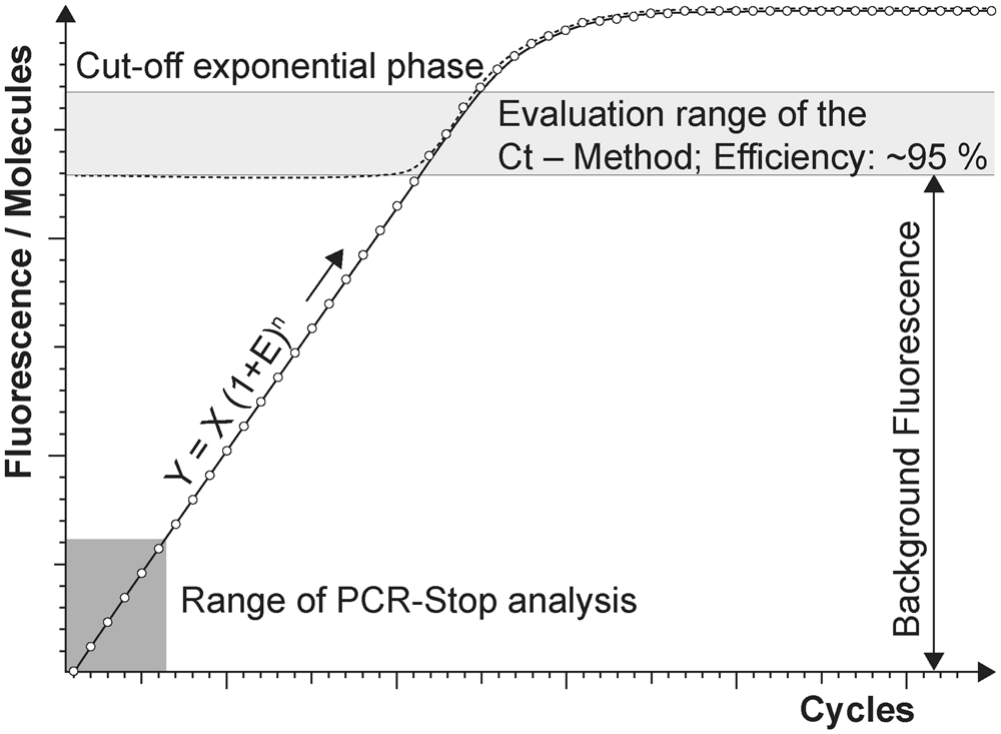
The real amplification curve of a qPCR assay. The range of observable and significant increase in fluorescence based on amplification of the PCR product is small. The exponential phase is mostly masked by the background fluorescence of the uncoupled probe dye. Therefore PCR-Stop analysis is an indirect approach depicting events during the first amplification cycles of qPCR.

The aim of PCR-Stop analysis is to improve understanding of qPCR. Firstly, the validation method should reveal assay performance in respect of amplification efficiency during initial cycles, which are otherwise invisible. The test system should show whether there is equal DNA duplication during initial cycles corresponding to overall efficiency as displayed in the calibration curve (Ct-Method). Consequently, it permits confirmation of the calculation using the formula y = x (1 + E)^n^ when qPCR starts with constant efficiency at the first cycle (Fig. 1; where y: copy number of the product at the end of a PCR reaction; n: cycles; E: efficiency; x: initial template copies). Further, knowing assay performance during first cycles permits Ct value prediction when one target copy is used as template (Ct_1_): In the case of 100% efficiency and a threshold equal to 10^10^ amplificates reflecting background fluorescence of unbound probe dye, one copy is equivalent to Ct_1_ = 37. Consequently, samples depicting Ct values significantly >37 lead to suspicion of inhibitory effects.

Secondly, PCR-Stop analysis is a validation tool for ranges >10 ITMN. It therefore ideally supplements Poisson analysis and reveals whether the assay has two-fold quantitative resolution. PCR-Stop analysis also discloses whether or not the polymerase reaction begins immediately with average efficiency and consequently whether or not the enzyme is completely activated. The latter point is especially useful for comparing hot start polymerases based on chemical modifications or those complexed with antibodies.

**Figure 2:**
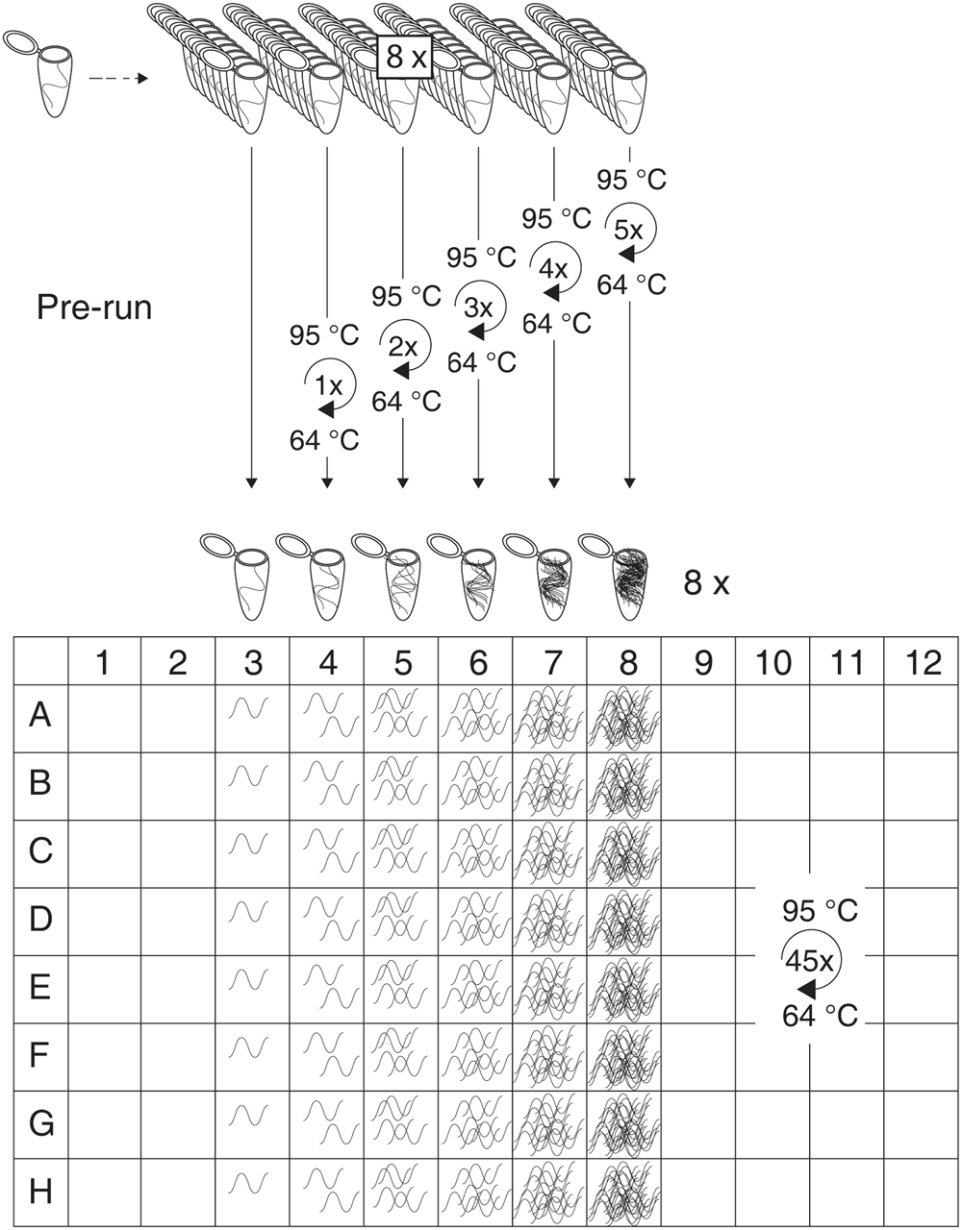
The experimental setup PCR-Stop analysis. Six batches each with eight samples containing the same target DNA quantity are subjected to up to five PCR pre-runs with ascending numbers of amplification cycles. Afterwards, all batches are amplified in a normal qPCR run (more details in the text).

**Figure 3:**
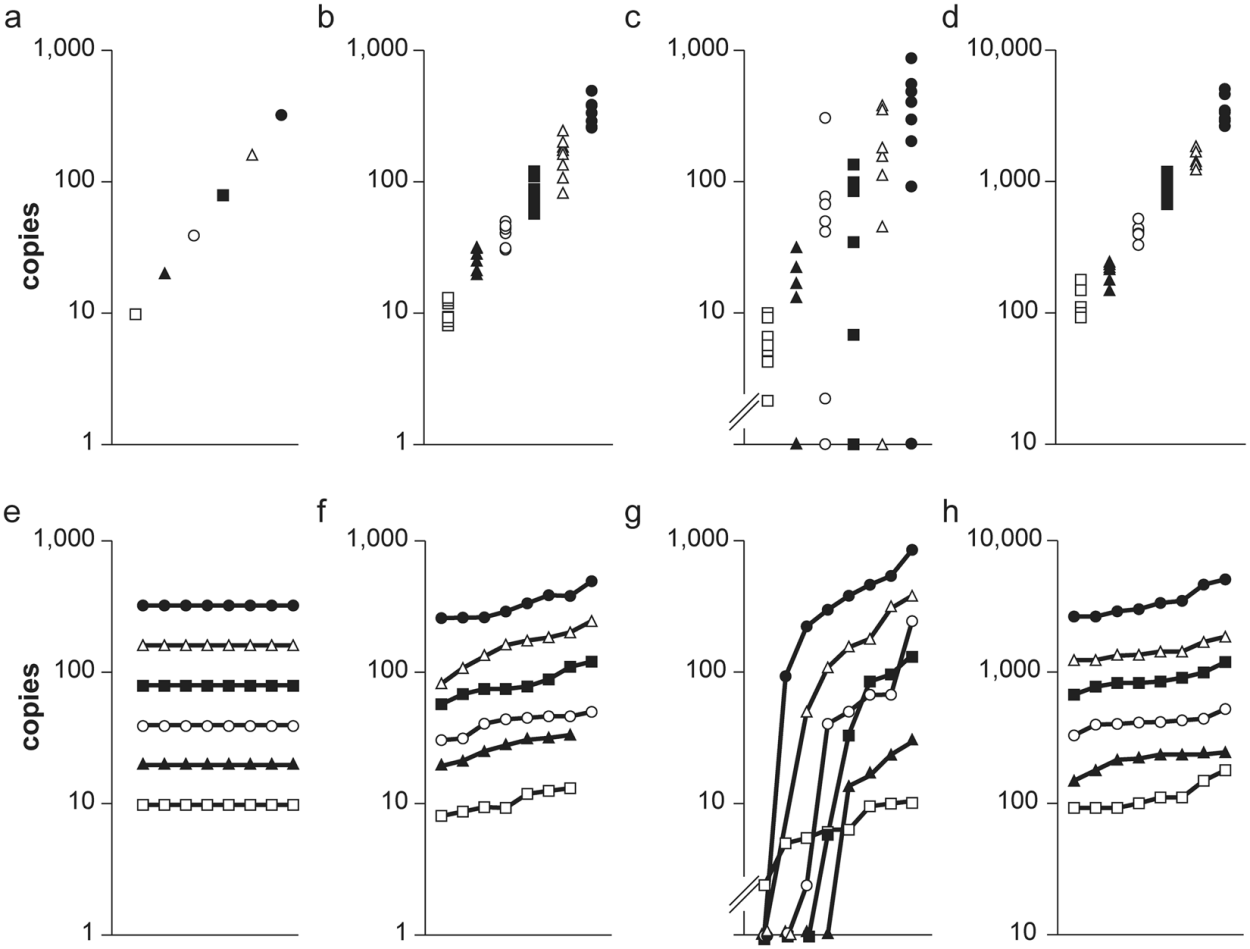
PCR-Stop analysis of a theoretical and two real qPCR assays. This illustrates two possibilities to determine the distribution within batches and the quantitative resolution of a theoretical optimal assay (**a**,**e**), the well performing *prfA* assay with 10 ITMN (**b**,**f**) and the *exB* with 10 (**d**,**h**) and 100 (**c**,**g**) ITMN. The ITMN are shown on the y-axis (**a**–**d**). In e-h the ITMN for the 48 samples are ordered by the quantities obtained after the final qPCR run. The eight batches are indicated in different dot styles (⦁, ∆, etc.). Not amplified samples (“No Ct”) are illustrated on the x-axis as.

The practical execution of PCR-Stop analysis is simple. It involves six batches, each including eight samples (ideally on a strip) containing the same target DNA quantity (exceeding the Poisson distribution; >10 ITMN). These batches are subjected to PCR pre-runs with ascending numbers of amplification cycles, starting from zero to five. When the first batch is directly placed into the cooler, the other batches are each subjected to short PCR runs, representing one to five cycles of the PCR assay to be tested, and later cooled. Subsequently all batches are transferred together to the real-time PCR thermo cycler for a normal run with the entire number of cycles (Fig. 2). These batches therefore represent ongoing amplification during the first five cycles. The first batch includes the original ITMN for each sample. The other batches should analogously represent the amplification according to the cycle numbers of the respective pre-runs. For a perfect qPCR assay with 100% efficiency this would lead to exact duplication of the ITMN during amplification. Moreover, no deviation among the single samples of each batch would be observed (Table 1, Fig. 3a,e). However, realistically, these values will deviate and the overall average efficiency will be less than 100%.

**Table 1:** Copy numbers and relative standard deviations (RSD) of different assays in the PCR-Stop analysis.

Pre-runs	Theory (10)		*prfA*(10)		*exB* (10)		*exB* (100)	
	AV (copies)	RSD (%)	AV (copies)	RSD (%)	AV (copies)	RSD (%)	AV (copies)	RSD (%)
0	10	0	11	20	7	63	117	27
1	20	0	26	19	3	282	220	16
2	40	0	42	17	97	103	426	13
3	80	0	87	24	21	174	878	17
4	160	0	153	31	57	201	1,440	15
5	320	0	310	25	318	58	3,456	26

AV indicates the average initial target DNA numbers for each batch containing eight samples. RSD indicates the relative standard deviation of the values within the eight samples of each batch. The perfect assay (Theory) shows 100% correspondence with the model prediction and 100% efficiency of the amplification. The well performing *prfA* assay in the range of 10 ITMN has a rather constant RSD or approximately 20%. The poorly performing *exB* assay for the range of 10 ITMN shows very high and inconsistent RDS, but nevertheless performs well (RSD ~20%) in the range of 100 ITMN.

PCR-Stop analysis can also be used for ITMN numbers significantly higher than the quantification limit of a given assay in order to test its quantitative resolution over a specific numerical range. In this layout the test offers the opportunity for testing assays using two-fold quantitative resolution.

### Analysis of PCR-Stop experiments and its practical application

For proof of principle, we performed the PCR-Stop experiment as described in the previous section using the *prfA* assay, which was prior validated with Poisson analysis^6,14^. Furthermore, this *prfA* assay was established for droplet digital PCR^15,16^. An unpublished *Salmonella enterica* serovar Typhimurium assay was used as second assay, hereafter referred to as *exB* (*example B*). Conventional analysis showed that efficiency (100.6%) and Rsq (0.998) from the calibration curve were sufficient (Supplemental Fig. S1), but Poisson analysis revealed slight underquantification (Supplemental Table S2). The average initial target DNA numbers containing eight samples for performing the PCR-Stop analysis are 10 (*prfA, exB*) or 100 ITMN (*exB*), respectively.

**Figure 4:**
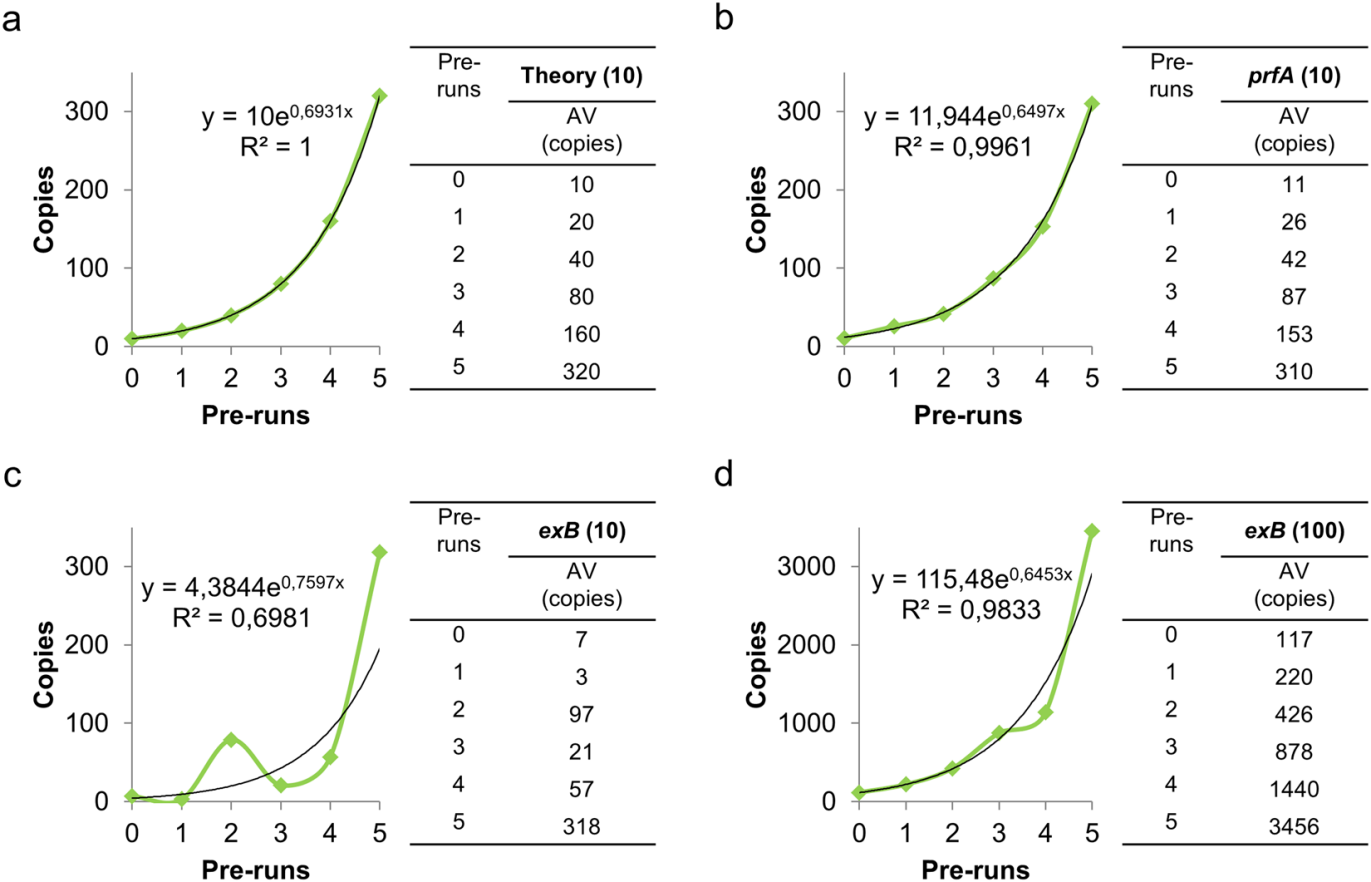
Efficiencies during the first cycles. The PCR-Stop experiment reveals efficiencies during the first five cycles of qPCR demonstrated in the theoretical perfect assay (**a**), *prfA* assay with 10 ITMN (**b**) and *exB* assay with 10 (**c**) and 100 (**d**) ITMN.

Four criteria can be determined for analysis of PCR-Stop experiments:

I. Duplication of the ITMN during pre-runs, demonstrating consistent efficiency of amplification during first cycles (Table 1),
II. the relative standard deviation within the values, obtained for the eight samples of each batch, demonstrating the consistency within the assay, as well as the qualitative limit if >10 ITMN (Table 1, Fig. 3),
III. the steady increase of values and regularity within the batches demonstrating quantitative assay resolution (Fig. 3e–h).
IV. The final criterion, namely negative samples representing the absence of amplification thereby demonstrating the qualitative limit if >10 ITMN (Fig. 3).

(I) The amplification efficiency during first cycles is calculated from the steady increase of the average value obtained for each batch including eight samples. This value is the first criterion for the quality of a given qPCR assay. In the *prfA* experiment presented, the efficiency was 93.7% when calculated with the PCR-Stop experiment and showed good correlation with the 94.6% efficiency obtained from the calibration curve. On the other hand, the efficiency of the *exB* assay was 100.6% as calculated from the calibration curve, but 109.6% using the PCR-Stop analysis with 10 ITMN and 93% with 100 ITMN. Further, the deviation of the optimal trend line was remarkable in the case of *exB* at 10 ITMN (R^2^ = 0.6981), while satisfactory with 100 ITMN (R^2^ = 0.9833), which demonstrated irregularity during initial cycles (Fig. 4). However, these R^2^ values demonstrate the irregularity of amplification and must not be confused with those Rsq values obtained with the Ct-method (see introduction).

(II) The second criterion, the relative standard deviation (RSD) of the values within the eight samples of each batch, is determined and then summarized in an average value for all batches (Table 1). A perfect assay would display 0% RSD. This value for the well performing *prfA* assay was approximately 20% RSD in all batches (Table 1). The RSD of *exB* even approached 300%. Nevertheless, the assay displays a deviation of approximately 20% RSD at higher target DNA concentrations (100 ITMN, Table 1), where the amplification reaction performs well. The lowest initial DNA target number at which this value of RSD is achieved thus represents the lower limit where good quantitative resolution for the given qPCR assay can be expected.

(III) Analysis of the third criterion displays the values of all 48 samples of the six batches summarized in graphs. For each batch in this case the values are ordered in an increasing sequence, resulting in six ascending graphs. For a perfect assay this would result in six horizontal lines with uniform distance (Fig. 3e). The graphs for the well performing *prfA* assay are illustrated in Fig. 3f. The graphs are evenly ascending, showing no overlap between batches. In contrast, overlaps are present in the *exB* assay using 10 ITMN (Fig. 3g), but with 100 ITMN the assay is again satisfactory (Fig. 3h). Figure a–d demonstrates the same values in an alternative graph revealing the performance of the quantitative resolution.

(IV) The occurrence of negative samples displaying lack of amplification during the PCR run indicate the qualitative detection limit of the given PCR assay, at the target DNA molecule number which was tested. This fourth criterion is significant if negative results accumulate and those samples are therefore identified as non-outliers. In this case the other three criteria will also display distinct deviations. In this case the experiment should be executed once more using a higher initial DNA quantity for determination of the quantitative limit and resolution. In case of the *exB* assay, regularly (in almost all batches at least one, Fig. 3) negative samples were present with 10 ITMN. This failure is in accordance with the other criteria which demonstrated deviating efficiency, high relative standard deviation and crossing lines in the respective graph of the *exB* assay.

## Discussion

Since the implementation of qPCR, numerous assays have been developed and are now used for a wide range of applications. However, it is frequently neglected that these enzymatic assays must be validated *per se*. Therefore, and in addition to the Poisson analysis, which covers the range <10 ITMN^6^, we developed PCR-Stop analysis, a method for assay validation covering >10 ITMN. This method enables: (1) assessment as to how an assay performs during the very first cycles, (2) quantitative resolution of the assay in the two-fold range, (3) determination of the lower limit of the reproduction/replication of a two-fold pattern, therefore determining the quantitative limit of the assay, (4) determination of the qualitative limit of detection if the amplification fails in a significant number of samples, and (5) calculation of the efficiency of the amplification reaction during the first cycles using the average results of several batches.

In 2004 Nogva and Rudi^17^ concluded that efficiency is lower during starting cycles. In contrast, we have been able to show with PCR-Stop analysis that the *prfA* assay already starts with its average efficiency. Results also demonstrate that the polymerase (in this special case Platinum Taq complexed with an antibody for the hot start property) is from the beginning active and completely functional. Interestingly, the *exB* assay also starts with a similar efficiency as the average efficiency from the calibration curve but the amplification is very irregular demonstrated by the low precision (R^2^) when only 10 ITMN were used.

Using the *exB* assay, we demonstrated that precise standard curves originating from serial dilutions with satisfactory efficiencies and Rsq values do not sufficiently displaying real assay performance. Despite 100.6% efficiency and an Rsq of 0.998, the PCR-Stop analysis for the *exB assay* revealed insufficient resolution in the boundary limit area. Therefore, this assay is not reliable for quantification in the ranges <100 ITMN. In practice, the obtained knowledge prevents misinterpretations of results in lower ranges.

qPCRs are now familiar in many applications with different emphases. For gene expression studies, which should also detect small differences (even lower than two-fold, e.g.^18^) qPCR is considered the *gold standard*^19^ needing normalization steps. However, the best normalization using proper reference genes^20^ and open-access tools for analysis^19^ etc., do not help when the resolution of the assay is not sufficient for the scientific question. The PCR-Stop analysis, together with Poisson experiment, reveals the real detection limit as well as the quantitative resolution of a qPCR assay. We therefore anticipate that these methods should be very helpful for such applications.

As also Kralik and Ricchi^21^ emphasised, appropriate validation improves qPCR quality. Consequently, we encourage other scientists to validate their assays with both the Poisson experiment and PCR-Stop analysis. Failing or performing poorly those demonstrate the limits of assays and reveal in which ranges assays are trustful and can be applied. These validations can thus assist assessment of qPCR results, avoid misinterpretations and achieve increased confidence with qPCR applications. The *MIQE* (Minimum Information for Publication of Quantitative Real-Time PCR Experiments) guidelines aim also at “more reliable and unequivocal interpretation of qPCR results”^4^ and therefore, the PCR-Stop analysis is in line with the guidelines. The information PCR-Stops analysis provides are thereby not considered and are thus are supplementing the category *assay performance* which includes efficiency, precision, etc.

## Methods

### DNA extraction and quantification

One ml of a *L*. *monocytogenes* (strain EGDe) *or Salmonella enterica* serovar Typhimurium (both part of the collection of bacterial strains at the Institute of Milk Hygiene, University of Veterinary Medicine, Vienna) overnight culture was used for DNA isolation using the NucleoSpin tissue kit (Macherey Nagel, Düren, Germany) following protocol instructions for Gram-positive or Gram-negative bacteria, respectively. The DNA was eluted twice with 50 μlddH_2_O (70 °C). The DNA concentration was measured with the Qubit ds Broad Range Kit (Fisher Scientific, Vienna, Austria). The copy number of the single-copy *prfA* gene was calculated using the molecular weight (1 ng of DNA equals 3.1 × 10^5^ copies of the genome of *L. monocytogenes*, 1 ng of DNA equals 1.9 × 10^5^ copies of the genome of *Salmonella enterica* serovar Typhimurium).

### qPCR

Real-time PCRs were performed as previously described^14^ in an Mx3000p real-time PCR thermocycler (Stratagene, CA, USA) and were analyzed using MxPro Software. One qPCR reaction (25 μl) contained 3.5 mM MgCl_2_, 12.5 pmol of each primer (prfA_fwd: 5′-GAT ACA GAA ACA TCG GTT GGC-3′; prfA_rev: 5′-GTG TAA TCT TGA TGC CAT CAG G-3′, Eurofins, Ebersberg, Germany), 6.25 pmol of each probe (prfA-probe: 5′-FAM-CAG GAT TAA AAG TTG ACC GCA-MGB-3′, Fisher Scientific, Vienna, Austria), 5 nmol each of dATP, dTTP, dGPT, and dCTP, 2.5 μl of 10× reaction buffer, 1.5 U of Platinum *Taq* DNA polymerase (Fisher Scientific, Vienna, Austria) and 5 μl template DNA. *prfA* qPCR was carried out with initial denaturation at 94 °C for 2 min following amplification in 45 cycles at 94 °C for 15 s, and 64 °C for 1 min. For *exB* qPCR, after initial denaturation at 95 °C for 5 min, amplification was performed in 50 cycles, at 95 °C for 5 s, and 60 °C for 1 min.

### PCR-Stop

After the pre-runs (according to the description in the text and in Table 2), the reactions were stored at 4 °C and qPCR was performed as described immediately after all pre-runs were finished.

**Table 2:** Pre-runs.

		Initial denaturation	Cycle	Cycle number
*prfA*	0	—	—	—
	1	94 °C, 2 min	94 °C, 15 s–64 °C 1 min	1
	2	94 °C, 2 min	94 °C, 15 s–64 °C 1 min	2
	3	94 °C, 2 min	94 °C, 15 s - 64 °C 1 min	3
	4	94 °C, 2 min	94 °C, 15 s–64 °C 1 min	4
	5	94 °C, 2 min	94 °C, 15 s–64 °C 1 min	5
*exB*	0	—	—	—
	1	95 °C, 5 min	95 °C, 5 s–60 °C 1 min	1
	2	95 °C, 5 min	95 °C, 5 s–60 °C 1 min	2
	3	95 °C, 5 min	95 °C, 5 s–60 °C 1 min	3
	4	95 °C, 5 min	95 °C, 5 s–60 °C 1 min	4
	5	95 °C, 5 min	95 °C, 5 s–60 °C 1 min	5

## Electronic supplementary material

Supplementary PCR-Stop analysis
