# Supplementary material for: PCR-Stop analysis as a new tool for qPCR assay validation
Source: Sci Rep. 2018 May 29;8:8275. doi: 10.1038/s41598-018-26116-x (PMC5974342; doi:10.1038/s41598-018-26116-x)
Supplement: Supplementary file 1 — Supplementary PCR-Stop analysis [file 41598_2018_26116_MOESM1_ESM.pdf]

Supplementary Information

PCR-Stop analysis as a new tool for qPCR assay validation

Anna Kristina Witte, Patrick Mester, Susanne Fister, Beate Süß, Martin Wagner and Peter Rossmanith

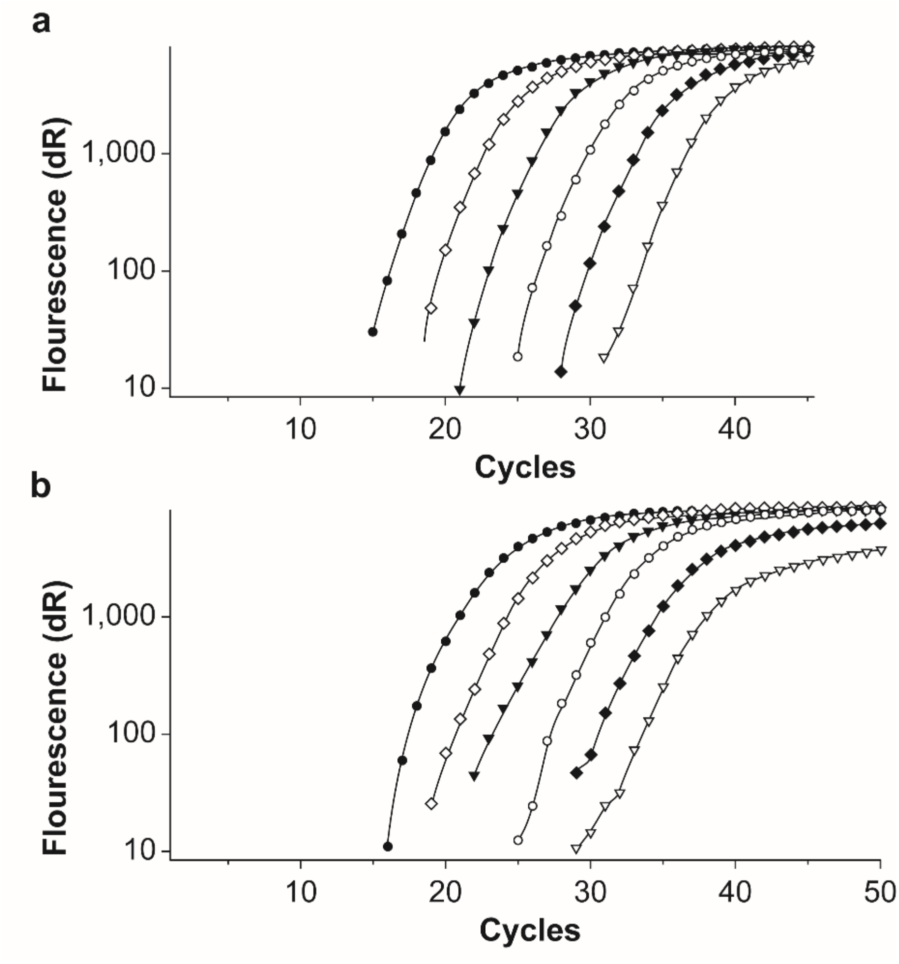

Supplemental Fig.S1: Standard curve of the *Listeria monocytogenes prfA* and *Salmonella enterica* serovarThyphiurium *exB* assays

Exemplary illustration of the *L. monocytogenes prfA* (a) and *S. enterica exB* qPCR showing a 10-fold serial dilution ranging from 1.58 x 10^6 to 15.8 ITMN with an efficiency of 94.7 % and an Rsq of 1.000 (a) or from 9.5 x 10^5 to 9.5 ITMN with an efficiency of 100.6 % and an Rsq of 0.999 (b), respectively.

Supplemental Table S2: Poisson analysis of *prfA* and *exB*

		1 copy	3 copies	10 copies	Conclusion
prfA	Average copies	0.96	3.0	10.6	Congruency ok
	Negative samples % (negative/total)	43 % (13/30)	7 % (2/30)	-	Fitting to theoretical expectations
	Theoretical expectation	40 %	5 %	-	
exB	Average copies	0.63	2.4	7.9	Congruency ok
	Negative samples % (negative/total)	56 % (17/30)	30 % (9/30)	-	Excess of negative samples (3 copies)
	Theoretical expectation	55 %	8 %	-	

One exemplary Poisson experiment of the *prfA* and *exB* are demonstrated showing average copy numbers and (relative and absolute) quantities of negative sample and the respective theoretical expectations. The *exB* assay has in the “3 copies” data set a distinct excess of negative samples leading to probable underquantification in data analysis.
